# Potential Biological Processes Related to Brain SLC13A5 Across the Lifespan: Weighted Gene Co-Expression Network Analysis from Large Human Transcriptomic Data

**DOI:** 10.3390/brainsci16020163

**Published:** 2026-01-30

**Authors:** Bruna Klippel Ferreira, Patricia Fernanda Schuck, Gustavo Costa Ferreira, Hércules Rezende Freitas

**Affiliations:** 1 Laboratório de Erros Inatos do Metabolismo, Programa de Bioquímica e Biofísica Celular, Instituto de Bioquímica Médica Leopoldo de Meis, Universidade Federal do Rio de Janeiro, Rio de Janeiro 21941-599, Brazil; bruna.klippel@bioqmed.ufrj.br (B.K.F.); patricia.schuck@bioqmed.ufrj.br (P.F.S.); 2 Laboratório de Informática em Saúde (LabInfoS), Departamento de Ciências Médicas Integradas, Faculdade de Ciências Médicas, Universidade do Estado do Rio de Janeiro, Cabo Frio 28905-320, Brazil

**Keywords:** SLC13A5, Na(+)/citrate cotransporter, developmental and epileptic encephalopathy, cerebrum

## Abstract

**Background/Objectives:** *SLC13A5* encodes a sodium–citrate cotransporter implicated in early-onset epileptic encephalopathy and metabolic brain dysfunction, yet its developmental regulation and molecular context in the human brain remain incompletely defined. **Methods:** Leveraging human developmental transcriptomes from the Evo-Devo resource, we delineated tissue trajectories and network context for *SLC13A5* across the fetal–postnatal life. **Results:** In the cerebrum, *SLC13A5* expression rises from late fetal stages to peak in the first postnatal year and then declines into adulthood, while cerebellar levels increase across the lifespan; liver shows a fetal decrease followed by sustained postnatal upregulation. A transcriptome-wide scan identified extensive positive and negative associations with *SLC13A5*, and a signed weighted gene co-expression network analysis (WGCNA) built on biweight midcorrelation placed *SLC13A5* in a large module. The module eigengene tracked brain maturation (Spearman rho = 0.802, *p* = 8.62 × 10^−6^) and closely matched *SLC13A5* abundance (rho = 0.884, *p* = 2.73 × 10^−6^), with a significant partial association after adjusting for developmental rank (rho = 0.672, *p* = 6.17 × 10^−4^). Functional enrichment converged on oxidative phosphorylation and mitochondria. A force-directed subnetwork of the top intramodular members (|bicor| > 0.6) positioned *SLC13A5* adjacent to a densely connected nucleus including *CYP46A1*, *ITM2B*, *NRGN*, *GABRD*, *FBXO2*, *CHCHD10*, *CYSTM1*, and *MFSD4A*. **Conclusions:** Together, these results define a developmentally tuned, mitochondria-centered program that co-varies with *SLC13A5* in the human brain across the lifespan. It may provide insights to interrogate age-dependent phenotypes and therapeutic avenues for disorders involving citrate metabolism.

## 1. Introduction

SLC13A5 epilepsy, also known as developmental epileptic encephalopathy 25 (OMIM # 615905), is an autosomal recessive disease caused by deficiency in SLC13A5 citrate transporter. The disease is characterized by neonatal seizures, febrile seizures, *status epilepticus*, developmental delay, a severe movement disorder, and lack of tooth enamel. Severe seizures start in the first days of life, with better seizure control in late childhood and adolescence but lifelong increased seizure risk [1]. Patients have global developmental delay and impaired motor function [2]. Tooth hypoplasia due to *amelogenesis imperfecta* remains a distinctive feature [3].

To date, more than 50 loss-of-function mutations in human *SLC13A5* have been found to cause SLC13A5 epilepsy [4,5]. Interestingly, there has been no genotype–phenotype correlation identified, though all tested mutations had a severe loss of citrate transporter function [6,7]. *Slc13a5*-knockout mice showed pro-epileptogenic neuronal excitability changes in the hippocampus, and approximately 50% of the mice had spontaneous seizures [8].

There are no curative treatments for SLC13A5 epilepsy, and all patients are treated with standard antiseizure medications, with mixed results. Previously reported antiseizure medications include benzodiazepines, phenobarbital, phenytoin, and carbamazepine, with good seizure control in some patients. However, some patients needed to use up to 10 drugs in polytherapy [1,2]. Although current antiseizure medications may reduce seizure frequency, more targeted treatments are needed to address the epileptic and non-epileptic features of SLC13A5 epilepsy, such as communication and movement disorders [9]. Additionally, SLC13A5 has been proposed as a molecular target for several diseases, such as metabolic syndrome, kidney disease, and cancer [10].

Studies have demonstrated that SLC13A5 epilepsy symptoms change with age [1,9]. However, it is unknown whether physiological SLC13A5 expression changes over time. The present work is therefore an effort to use large transcriptomic data to investigate SLC13A5 expression in humans.

## 2. Materials and Methods

### 2.1. Data Sources

The Evo-Devo application, created by Cardoso-Moreira et al. (2019) [11], is a vast database including expression results for genes in different species, organs, and development stages. Human RNA-seq expression (RPKM) across developmental stages was obtained from the Evo-Devo resource [11]. *SLC13A5* and genome-wide expression for brain and peripheral tissues reported by Evo-Devo were analyzed. Analyses focused on *Homo sapiens* only. Descriptive trajectories were assembled for brain, cerebellum, kidney, liver, testis, and ovary.

### 2.2. Preprocessing and Sample Ordering

Expression tables were reshaped into sample-by-gene matrices keyed by Ensembl gene identifiers. To stabilize variance, values were transformed as log2(RPKM + 1). Genes with zero variance or entirely missing values were removed, and Ensembl version suffixes were stripped to harmonize identifiers. Cerebrum samples were arranged according to a biologically consistent developmental sequence spanning from 4 to 20 weeks post-conception (wpc) through newborn, infant (6 to 9 months old), toddler (2 to 4 years old), school age (7 to 9 years old), teenager (13 to 19 years old), young adult (25 to 32 years old), young mid-age (39 to 41 years old), older mid-age (46 to 54 years old), and senior (58 to 63 years old). Since the Evo–Devo developmental labels are ordinal rather than metrically spaced, an ordinal trait (age rank) was constructed by assigning ranks from 1 to N along this sequence. Data quality was assessed using the WGCNA goodSamplesGenes criterion, with a minimum non-missing fraction of 0.30 [12]; only samples and genes passing quality control were retained.

### 2.3. Transcriptome-Wide Association with SLC13A5

Within the brain data set, transcriptome-wide association was performed across cerebrum samples, correlating *SLC13A5* (Stable ID: ENSG00000141485) with each expressed gene by using Spearman rank correlation and pairwise handling of missingness. Genes present in at least 80% of samples were included in the correlation analysis. Transcriptome-wide developmental correlation screening used 22 developmental samples and 37,743 genes, yielding 830,346 sample × gene measurements in the analyzed expression matrix. Downstream enrichment analyses used this same set of 37,743 tested genes as the background universe, with a significant input set of 13,915 genes at FDR < 0.05. Two-sided P-values were adjusted for multiple testing using Benjamini–Hochberg false discovery rate (FDR). The full correlation landscape was summarized with a volcano-type display and a compact temporal heatmap of the top positively and negatively associated genes to visualize developmental coherence.

### 2.4. Weighted Gene Co-Expression Network Analysis (WGCNA)

A signed co-expression network was constructed from the brain matrix using biweight midcorrelation (bicor). The soft-thresholding power was chosen from the range of 1–20 as the first value, achieving a scale-free topology fit of R^2^ ≥ 0.80; when no value reached this criterion, a conservative default of 6 was used [13]. Modules were identified with blockwise hierarchical clustering using signed topology overlap, a minimum module size of 30 genes, a merge cut height of 0.25, zero reassignment threshold, and partitioning around medoids respecting the dendrogram [14]. For each module, the first principal component (module eigengene) was computed to summarize expression.

### 2.5. Module–Trait and Gene–Module Relationships

Associations between the *SLC13A5* module eigengene (SME) and developmental progression were tested using Spearman correlation with the ordinal age group rank. The relationship between SME and *SLC13A5* expression was assessed analogously. A partial Spearman association between SME and *SLC13A5* controlling for age rank was obtained by correlating rank-based residuals from linear models. Intramodular connectivity (kME) was quantified as the signed correlation between each gene and its own module eigengene, providing a continuous measure of hubness.

### 2.6. Functional Enrichment

Functional enrichment for the *SLC13A5* module was performed while keeping gene-identifier universes consistent with each analysis. Gene Ontology Biological Process testing used Ensembl IDs for both input and background (all genes passing network quality control), with FDR control by Benjamini–Hochberg [15]. KEGG analysis required mapping Ensembl to Entrez Gene identifiers; both the module and the background were mapped symmetrically with deduplication at the Entrez level prior to testing, and FDR was controlled analogously. Transcription factor target enrichment used MSigDB C3 TFT signatures retrieved via msigdbr [16], tested as over-representation on HUGO Gene Nomenclature Committee (HGNC) symbols with a matching symbol-level background. Enrichment results were summarized by −log10(FDR) and gene-ratio for the most significant terms.

### 2.7. Co-Expression Subnetwork Visualization

To illustrate intramodular organization, the 30 genes with the highest absolute kME within the *SLC13A5* module were selected, with *SLC13A5* forcibly included (if not originally among the top 30). Pairwise bicor values were computed within this set, and an undirected edge was drawn when the absolute correlation exceeded 0.60 [17]. The graph was laid out with a Fruchterman–Reingold force-directed algorithm using a fixed random seed for reproducibility. Node size and color encode |kME|, labels are shown for *SLC13A5* and the highest-connectivity genes, and edges incident to *SLC13A5* are highlighted to delineate its immediate neighborhood.

### 2.8. Statistical Considerations

Since expression distributions deviated substantially from normality, nonparametric measures, such as Spearman’s rho (ρ), were used throughout for association. All tests were two-sided, and multiplicity was controlled by Benjamini–Hochberg FDR unless otherwise stated. Random seeds were fixed where stochastic procedures were involved to ensure reproducibility of visual layouts and summaries. All analyses were performed using the R language (version 4.5.1) with IDE RStudio (version 2025.9.1.401). A reproducible script is provided as Supplementary Material.

## 3. Results

**Figure 1:**
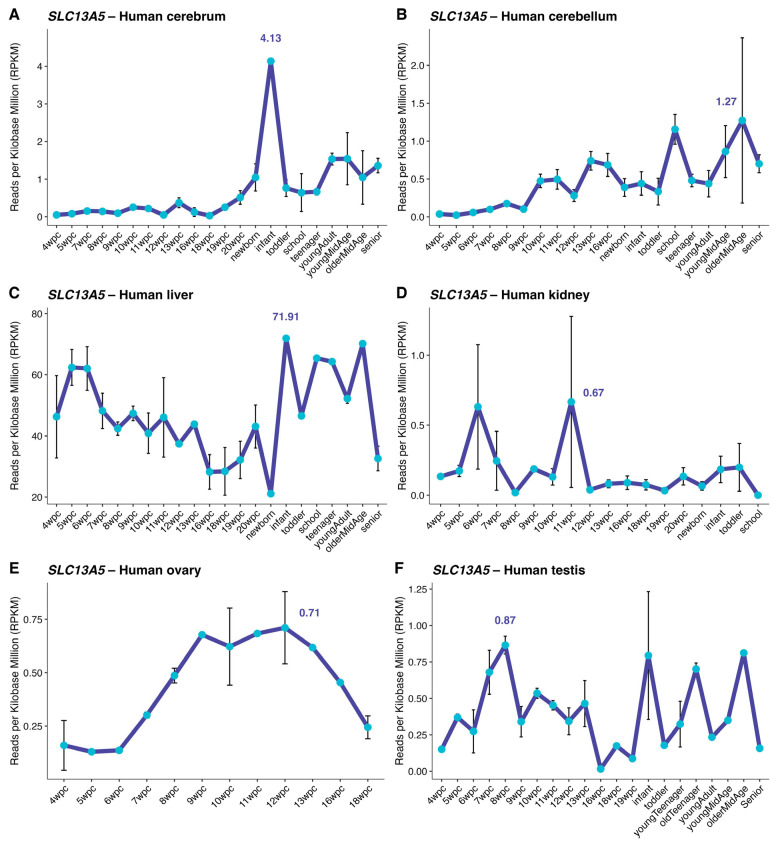
*SLC13A5* expression in human central and peripheral tissues. RPKM for *SLC13A5* expression is shown at different age categories for (**A**) cerebrum, (**B**) cerebellum, (**C**) liver, (**D**) kidney, (**E**) ovary, and (**F**) testis. Each panel depicts the biological ordering from fetal weeks post-conception (wpc) through postnatal stages, with the within-tissue median ± IQR annotated. The horizontal axis represents developmental progression through age ranks, including 4–20 wpc, newborn, infant (6 to 9 months old), toddler (2 to 4 years old), school age (7 to 9 years old), teenager (13 to 19 years old), young adult (25 to 32 years old), young mid-age (39 to 41 years old), older mid-age (46 to 54 years old), and senior (58 to 63 years old). Data are expressed as mean RPKM ± SEM.

To determine *SLC13A5* expression across tissues and how expression changes throughout development, we analyzed *SLC13A5* expression using the human data from Cardoso-Moreira et al. (2019) [11]. Figure 1 shows the longitudinal view of *SLC13A5* expression in different human tissues, namely cerebrum (Figure 1A), cerebellum (Figure 1B), liver (Figure 1C), kidney (Figure 1D), testis (Figure 1E), and ovaries (Figure 1F). Mean values for pre- and post-conception *SLC13A5* expression (RPKM) by tissue are shown in Table 1 (raw data available in Supplementary Data S1).

In the cerebrum, *SLC13A5* expression increases from ~0.5 RPKM at 19 wpc to ~4 RPKM in the first year of life. This is followed by a decrease in cerebrum *SLC13A5* expression until adult life, when levels are kept above 1 RPKM for the following decades. In early stages of development, cerebellar *SLC13A5* expression is less than 1 RPKM (0.3 *±* 0.3 RPKM) but increases slowly and continuously throughout life (0.7 *±* 0.3 RPKM). Liver is the tissue with the highest *SLC13A5* expression. Following an initial drop during the fetal period until birth (from ~50 RPKM to ~20 RPKM), *SLC13A5* expression increases and is kept high (~60 RPKM) until the end of adulthood. Other peripheral tissues (including kidney, ovaries, and testis) are also low throughout life.

**Figure 2:**
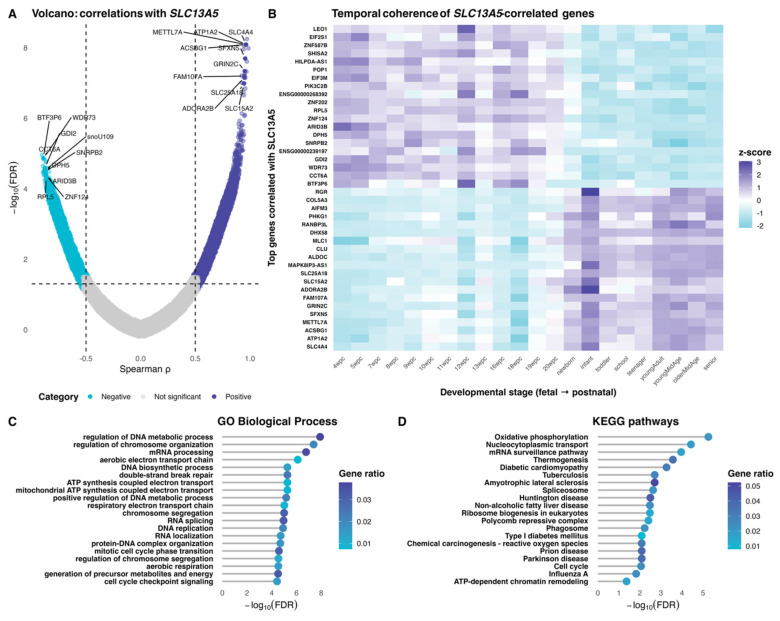
Transcriptome-wide association landscape for *SLC13A5* in the human cerebrum. Panel (**A**) shows a volcano-type display of Spearman correlations between *SLC13A5* and all expressed genes across cerebrum samples (dashed guides at ρ = ±0.5 and at the Benjamini–Hochberg FDR = 0.05 threshold). Labeled points indicate the most significant positive and negative associates after FDR correction. Panel (**B**) presents a compact heat map of the top positively and negatively associated genes (at all developmental stages from 4 wpc to senior), showing z-scored expression to emphasize temporal gradient. Panels (**C**,**D**) summarize functional enrichment among FDR-significant correlates: (**C**) Gene Ontology Biological Process using Ensembl identifiers for *SLC13A5*-correlated genes; (**D**) KEGG pathways using a symmetric Ensembl → Entrez mapping. The color of the dots indicates the gene ratio. The *y*-axis reflects −log10(FDR), with terms ranked by significance.

We then assessed the genes whose expression co-varies with *SLC13A5* across development in the human cerebrum transcriptome. A transcriptome-wide correlation scan across Evo-Devo cerebrum samples revealed extensive bidirectional associations with *SLC13A5* expression (Figure 2A, Supplementary Data S2). It includes both positively and negatively correlated genes after multiple-testing correction. To examine whether these relationships are developmentally organized, we evaluated z-scored expression for the top *SLC13A5*-correlated genes across fetal-to-postnatal stages. The heat map shows temporal coherence, with many transcripts mirroring the fetal-to-postnatal shift observed for *SLC13A5* (Figure 2B). Over-representation analyses of this set of genes flagged processes/pathways linked to mitochondrial energy metabolism (notably oxidative phosphorylation), RNA processing and surveillance (including spliceosome-related terms), and cell-cycle/chromatin regulation (Figure 2C,D).

**Figure 3:**
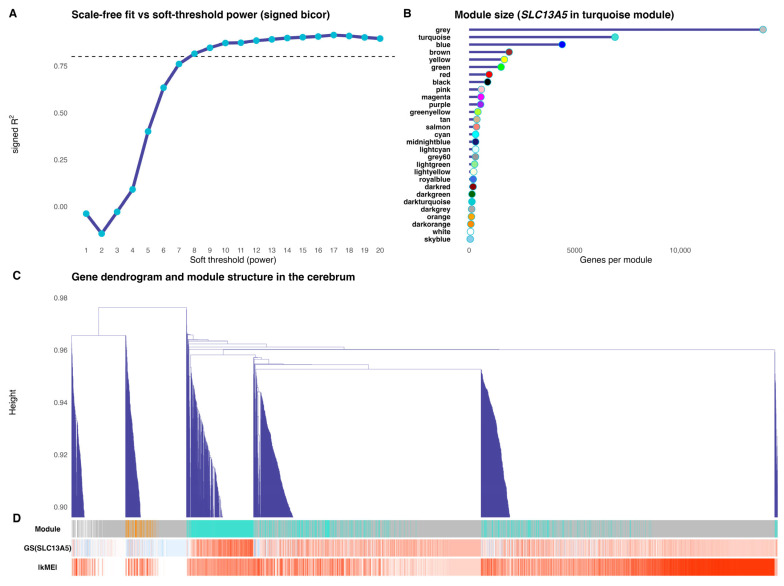
Network architecture of the cerebrum co-expression map. Panel (**A**) displays the scale-free topology fit versus soft-thresholding power for a signed, bicor network, with a chosen beta of 8, corresponding to the first value achieving R^2^ ≥ 0.80 (fallback applied when no value reaches the criterion). Panel (**B**) shows module size distribution (genes per module), with the circle color fill matching the WGCNA module color key used throughout. Panels (**C**,**D**) show the gene dendrogram with aligned color tracks: (**C**) dendrogram zoomed at the top to emphasize branch topology; (**D**) annotation tracks reporting, in order, module assignment (one block showing genes represented by their module color, according to panel B), gene significance to *SLC13A5* (bicor with *SLC13A5*; blue[high]-white-red[low] gradient), and intramodular connectivity (|kME|; blue[high]-white-red[low] gradient).

Network construction of the human cerebrum yielded a scale-free-like topology at low double-digit soft thresholds, with the signed bicor fit approaching the conventional R2 = 0.8 plateau and remaining stable thereafter (Figure 3A). Using this parameter, WGCNA identified a heterogeneous module landscape with a few very large groups and many smaller ones (Figure 3B); the “grey” set aggregated unassigned genes, while the turquoise and blue modules comprised the largest structured clusters. The hierarchical dendrogram revealed block structure (Figure 3C). The aligned annotation tracks (Figure 3D) showed that *SLC13A5* is found in the turquoise module, where genes with the strongest gene significance to *SLC13A5* (red in GS track) spatially co-localize with high intramodular connectivity (deep red in |kME| track).

**Figure 4:**
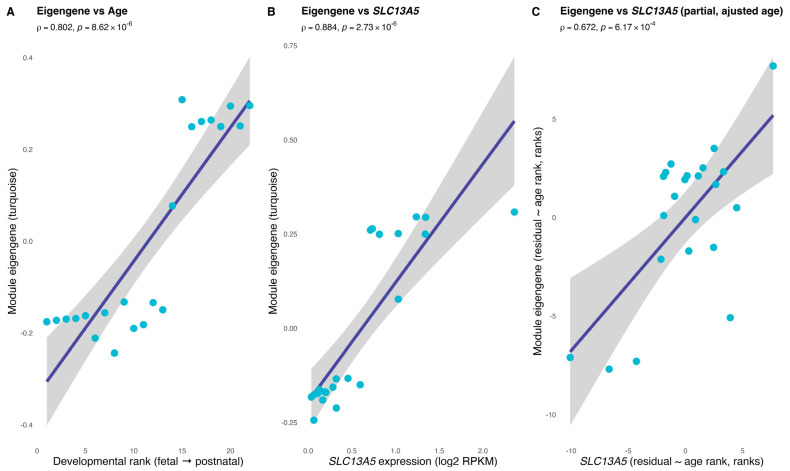
The *SLC13A5* module tracks neurodevelopment and the gene itself. Panel (**A**) shows the association between the *SLC13A5* module eigengene (SME) and the ordinal developmental rank (age rank) across cerebrum samples. Panel (**B**) depicts the association between SME and *SLC13A5* expression (log2-transformed). A partial Spearman correlation controlling for age rank (computed on rank-based residuals) is provided in the panel (**C**). Spearman ρ and two-sided *p* are indicated in all panels.

The *SLC13A5* module’s eigengene tracked cerebrum maturation and the gene’s own expression (Figure 4). Across fetal-to-postnatal stages, the eigengene rose in accordance with developmental rank (Spearman rho = 0.802, *p* = 8.62 × 10^−6^; Figure 4A), indicating that the turquoise module is progressively activated during human cerebrum development. The eigengene was also tightly correlated with *SLC13A5* expression itself (rho = 0.884, *p* = 2.73 × 10^−6^; Figure 4B), consistent with *SLC13A5* being embedded within, and representative of, the module. Importantly, this association persisted after regressing out age effects: A partial correlation between the eigengene and *SLC13A5* (controlling for developmental rank) remained significant (rho = 0.672, *p* = 6.17 × 10^−4^; Figure 4C).

**Figure 5:**
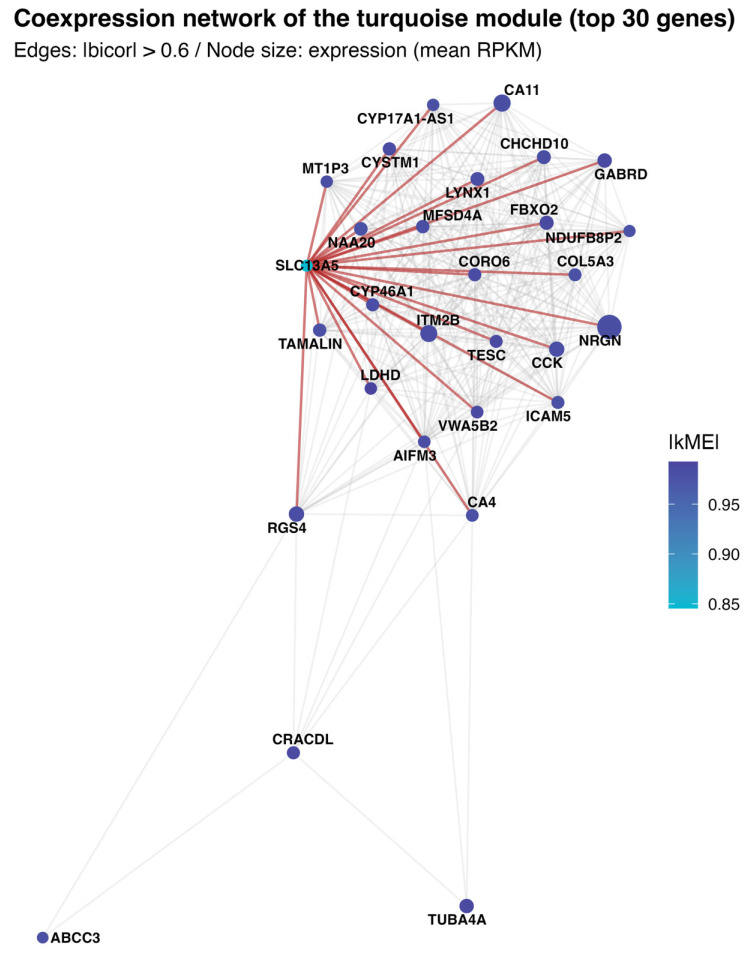
Intramodular co-expression network linked to *SLC13A5*. A force-directed graph depicts the top 30 genes by absolute intramodular connectivity (|kME|) within the *SLC13A5* module, with *SLC13A5* forcibly included when necessary. Undirected edges connect pairs with |bicor| > 0.60, emphasizing robust associations. Node color encodes |kME|, and size represents cerebrum gene expression (mean RPKM); labels are shown for *SLC13A5* and the highest-connectivity nodes. Edges incident to *SLC13A5* are highlighted (in red) to delineate its immediate neighborhood within the module.

To visualize the local wiring of the *SLC13A5* module, a force-directed subnetwork composed of the top 30 module members was plotted (ranked by |kME|); edges represent robust pairwise co-expression (|bicor| > 0.6) (Figure 5). The layout reveals a compact nucleus of highly interconnected genes with high module cohesion, flanked by a few peripheral nodes with weaker within-module connectivity. *SLC13A5* sits adjacent to the core and forms numerous strong links to neuronal and mitochondrial/transport genes (including *CYP46A1*, *ITM2B*, *NRGN*, *GABRD*, *FBXO2*, *CHCHD10*, *CYSTM1*, *MFSD4A*, *CORO6*, and *LYNX1*), consistent with the functional enrichments for oxidative metabolism, RNA/protein homeostasis, and synaptic programs. In contrast, nodes such as *ABCC3*, *CRACDL*, and *TUBA4A* occupy a more peripheral position with fewer edges, indicating lower intramodular connectivity.

**Figure 6:**
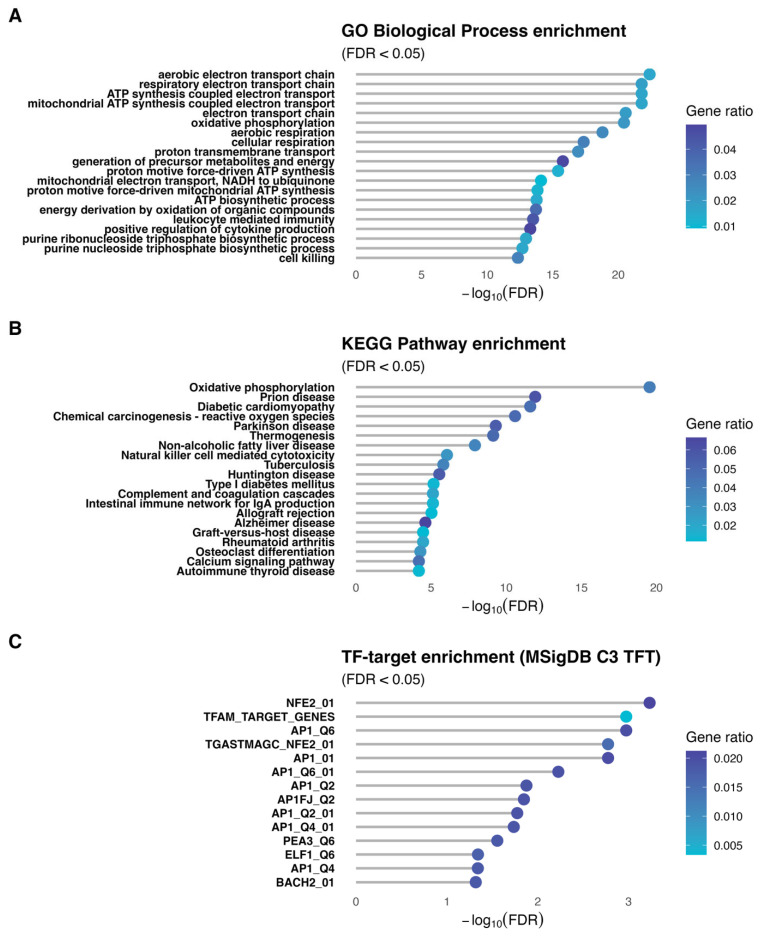
Functional programs enriched in the SLC13A5 module. Gene set over-representation for the *SLC13A5* module is shown for Gene Ontology Biological Process (Panel (**A**)), KEGG pathways (Panel (**B**)), and MSigDB C3 transcription factor targets (Panel (**C**)). Universes are matched to each test (all network-tested genes in Ensembl for GO; symmetric Ensembl → Entrez mapping for KEGG; symbol-level background for TF targets). Points encode the gene ratio by color, and the y-axis indicates −log10(FDR). Displayed terms are the top results by FDR, highlighting processes and pathways linked to the module’s coordinated variation.

Gene set enrichment of the *SLC13A5*-containing module revealed a coherent, mitochondria-centered program (Figure 6). In GO Biological Process (Figure 6A), top terms were related to mitochondrial metabolism, including ‘aerobic electron transport chain’, ‘respiratory electron transport chain’, ‘ATP synthesis coupled electron transport’, and ‘mitochondrial ATP synthesis coupled electron transport’. Other terms also revealed involvement with inflammation and purine metabolism. KEGG analysis (Figure 6B) showed a significant enrichment for ‘oxidative phosphorylation’ and pathways involved in neurodegenerative and inflammatory diseases. Transcription factor target enrichment (MSigDB C3 TFT; Figure 6C) highlighted regulators consistent with these themes, including NFE2/NRF-like motifs, TFAM-associated genes, and AP-1 family targets (multiple AP1 motif sets), as well as ELF1/BACH2 target sets.

## 4. Discussion

SLC13A5 plays a key role in citrate metabolism, impacting hepatic lipogenesis, cell proliferation, bone development, and epilepsy in mammals [18]. Loss-of-function mutations in the *SLC13A5* gene have been associated with SLC13A5 epilepsy [19]. On the other hand, overexpression of *Slc13a5* in neurons from mouse forebrain has been linked to disrupted white matter integrity and autistic-like behaviors [20]. *Slc13a5* overexpression also causes progeria-like phenotype, systemic inflammation, and alterations in protein acetylation [21]. Thus, SLC13A5 may present different roles/importance across the lifespan.

The physiological pattern of *SLC13A5* expression across life in human tissues has not been described yet. We therefore used a dataset of human tissues from Moreira and colleagues (2019) [11] to start addressing this issue. Liver was the tissue with the highest *SLC13A5* mRNA expression at all timepoints investigated. Previous reports showed *SLC13A5* expression to be much higher in liver than in brain (for both humans and rats) [22,23]. In cerebrum, *SLC13A5* mRNA expression increased from conception until infancy, when it reached its peak. An increase in *Slc13a5* mRNA expression during early postnatal life was also shown in rat cerebral cortex [24]. Cerebellar expression of *SLC13A5* steadily increased throughout life. *Slc13a5*-knockout mice show distinct metabolic pathways disrupted depending on the tissue investigated [25]. Thus, the distinct patterns of *SLC13A5* mRNA expression reported here may cooperate with the different roles played by SLC13A5 in the metabolism of these tissues.

We then assessed expression data of all cerebrum genes in the Evo-Devo database and ran multiple Spearman correlation analyses against the longitudinal expression of *SLC13A5*. We found sets of genes with strong correlation (positive or negative) and time coherence appropriateness with *SLC13A5* expression. Analysis of gene ontology and KEGG pathways indicates that *SLC13A5* sits in a developmentally coherent gene neighborhood enriched for mitochondrial bioenergetics and gene regulatory pathways in the human cerebrum. Genes related to transcription, translation, and synthesis of proteins are critical during neurodevelopment, and dysfunction of these genes may cause reduced brain volume, developmental delay, cognitive deficits, alterations in neural cristae, and neuronal alteration [26,27,28,29]. Additionally, the tuning of bioenergetic metabolism is crucial during neurodevelopment. Shifts in bioenergetics control cell fate, as well as neural progenitor proliferation and differentiation [30].

In order to evaluate the network hierarchy, we performed a weighted gene co-expression network analysis and evaluated the intramodular connectivity. The results of WGCNA validated the chosen network parameters, delineated the global co-expression architecture of the developing brain, and defined a densely connected *SLC13A5*-centered module for downstream analyses. The analyses of association between *SLC13A5*, the eigengene, and the developmental rank indicated that the module captures a coordinated expression program that matches developmental progression and specifically co-varies with *SLC13A5* (regardless of the global age trajectory).

The analysis of |kME| within the *SLC13A5* module suggested that *SLC13A5* is embedded in a densely connected module. It is directly connected to genes involved in neuronal homeostasis (synaptic signaling and structural proteins), mitochondrial organization, and lipid/cholesterol turnover. It is feasible that SLC13A5 plays a role in the cooperation between brain and liver for citrate homeostasis, maintaining lipid balance throughout the body (including basic and complex lipids) [25]. We also observed the presence of genes important for calcium handling (e.g., *NRGN* and *TESC*) [31,32,33] for cell signaling, metabolism, and fate (e.g., *CYP46A1*, *LDHD*, *CA4*, *CA11*, *RGS4*, *CHCHD10*, *AIFM3*, and *TAMALIN*) [34,35,36,37,38,39,40], as well as cell structure and extracellular matrix (e.g., *COL5A3*, *ICAM5*, *TUBA4A*, and *CORO6*) [41,42,43,44]. Interestingly, *GABRD* was found to be directly connected to *SLC13A5* in the module. *GABRD* is a gene that encodes GABA_A_ subunit, an important receptor during neurodevelopment and for epilepsy [45]. *SLC13A5* was also directly connected to *LYNX1*, a gene encoding a protein that modulates nAChR. Alterations in nAChR are associated with some epilepsies [46]. *LYNX1* dysregulation was reported in Fragile X Syndrome, a condition characterized by epilepsy [47], and in neurodevelopmental disorders [48]. Additionally, knockout of *Lynx1* in animal models enhances synaptic efficacy and performance in memory tests. However, it induces neurodegeneration by the hyperactivation of nAChR [49].

The functional programs enriched in the *SLC13A5* module were then analyzed. GO Biological Process enrichment analysis indicated that the *SLC13A5* module is embedded in a developmental program with genes involved in mitochondrial energy metabolism, reflecting a transcriptional coordination between mitochondrial homeostasis and *SLC13A5*. KEGG analysis showed an enrichment in pathways associated with neurodegenerative disease (Parkinson’s, Alzheimer’s, Huntington’s disease) and metabolic and inflammatory diseases (type I diabetes, non-alcoholic fatty liver disease, rheumatoid arthritis). Interestingly, SLC13A5 inhibition has also been suggested as a potential therapeutic target for kidney disease [50], hyperlipidemia [51], non-alcoholic fatty liver disease, insulin resistance, and a myriad of metabolic diseases [10,52,53,54]. The underlying mechanisms may involve decreasing citrate uptake from blood and reducing intracellular levels of citrate in the liver [55]. The enriched transcription-factor targets include factors such as NFE2, TFAM, and members of the AP-1 family (API, AP1F), implicated in the regulation of mitochondrial biogenesis, stress response, and cell differentiation [42,56,57,58]. The presence of TFAM, a key regulator of mitochondrial transcription, is particularly relevant. Altogether, the data suggest that this module may represent a regulatory axis relevant to physiological and pathophysiological conditions involving mitochondria.

It is important to emphasize that our analyses rely on transcriptomic data quantified as RPKM, which does not always correlate directly with protein abundance or functional activity. In fact, mRNA levels explain only about 40% of the variability in protein abundance across human tissues, highlighting the inherent constraints of using transcriptomic data alone to infer protein expression or biological function [59,60]. Therefore, protein abundance and localization must be addressed with orthogonal data. Moreover, potential confounders such as age-dependent changes in cell type composition, inter-donor variability, sex, and technical artifacts were not systematically addressed in this study. These factors may influence both transcript and protein measurements and should be considered when interpreting the data. While our analyses focus on within-series co-variation and use rank-based associations with matched testing universes to mitigate some sources of bias, we cannot exclude the possibility that part of the reported pathway enrichment and co-expression structure is influenced by these factors.

## 5. Conclusions

*SLC13A5* is highly expressed in the brain in the first years of life, suggesting an important role in this period of life and coinciding with the onset of seizures in SLC13A5 epilepsy patients. Potential targets of metabolic interplay with SLC13A5 include mitochondria, neurotransmission-related genes, and lipid metabolism. These findings deepen our understanding of the *SLC13A5* expression patterns and highlight its potential significance in cellular metabolism and disease pathogenesis. Continued investigation into the molecular mechanisms underlying SLC13A5 regulation and its functional implications in health and disease will be essential for unraveling its full biological significance and therapeutic potential. For instance, a better understanding of the mechanisms behind age- and tissue-specific *SLC13A5* transcription would help identify targeted therapeutics for SLC13A5 epilepsy and other metabolic disorders with altered citrate homeostasis. Future studies should also investigate neurotransmitter changes after loss of SLC13A5 to elucidate the functional role of SLC13A5 in neurotransmission.

## Figures and Tables

**Table 1:** Pre- and post-conception *SLC13A5* expression (RPKM) by tissue.

			Conception Stage				
Tissue	nDS	Overall ^1^	Pre ^1^	Post ^1^	Diff. ^2^	95% CI ^2^	*p*-Value ^2^
Cerebrum	22	0.7 ± 0.9	0.2 ± 0.1	1.4 ± 1.1	−1.2	−2.1, −0.40	0.009
Cerebellum	20	0.5 ± 0.4	0.3 ± 0.3	0.7 ± 0.3	−0.39	−0.69, −0.09	0.015
Heart	19	0.1 ± 0.1	0.0 ± 0.1	0.1 ± 0.1	−0.01	−0.12, 0.09	0.8
Kidney	18	0.2 ± 0.2	0.2 ± 0.2	0.1 ± 0.1	0.08	−0.08, 0.24	0.3
Liver	22	46.9 ± 14.2	43.5 ± 10.3	53.0 ± 18.6	−9.5	−26, 6.4	0.2
Ovary	12	0.4 ± 0.2	0.4 ± 0.2	-	-	-	-
Testis	21	0.4 ± 0.3	0.4 ± 0.2	0.4 ± 0.3	−0.08	−0.33, 0.18	0.5

^1^ Mean ± SD; ^2^ Welch two-sample *t*-test; abbreviation: CI = confidence interval, nDS = number of developmental stages available in the dataset, Diff. = diference between pre and post conception stage.

## Data Availability

The raw data supporting the conclusions of this article will be made available by the authors upon reasonable request. Evo-Devo resource website: https://apps.kaessmannlab.org/evodevoapp/ (accessed on 6 July 2023).

## Supplementary Materials

The following supporting information can be downloaded at: https://www.mdpi.com/article/10.3390/brainsci16020163/s1, a reproducible script (Script1) is provided as supplementary material, Data S1: Mean developmental SLC13A5 expression in human tissues, Data S2: Genes correlated to SLC13A5 throughout development.
